# *Perilla frutescens* Extracts Protects against Dextran Sulfate Sodium-Induced Murine Colitis: NF-κB, STAT3, and Nrf2 as Putative Targets

**DOI:** 10.3389/fphar.2017.00482

**Published:** 2017-08-08

**Authors:** Deung Dae Park, Hye-Won Yum, Xiancai Zhong, Seung Hyeon Kim, Seong Hoon Kim, Do-Hee Kim, Su-Jung Kim, Hye-Kyung Na, Atsuya Sato, Takehito Miura, Young-Joon Surh

**Affiliations:** ^1^Tumor Microenvironment Global Core Research Center, Seoul National University Seoul, South Korea; ^2^Department of Molecular Medicine and Biopharmaceutical Sciences, College of Pharmacy, Seoul National University Seoul, South Korea; ^3^Cancer Research Institute, Seoul National University Seoul, South Korea; ^4^Department of Food Science and Biotechnology, College of Knowledge-Based Services Engineering, Sungshin Women’s University Seoul, South Korea; ^5^Amino Up Chemical Co., Ltd. Sapporo, Japan

**Keywords:** colitis, dextran sulfate sodium, inflammatory bowel disease, *Perilla frutescens*, NF-κB, STAT3

## Abstract

*Perilla frutescens* is a culinary and medicinal herb which has a strong anti-inflammatory and antioxidative effects. In the present study, we investigated the effects of *Perilla frutescens* extract (PE) against dextran sulfate sodium (DSS)-induced mouse colitis, an animal model that mimics human inflammatory bowel disease (IBD). Five-week-old male ICR mice were treated with a daily dose of PE (20 or 100 mg/kg, *p.o.*) for 1 week, followed by administration of 3% DSS in double distilled drinking water and PE by gavage for another week. DSS-induced colitis was characterized by body weight loss, colon length shortening, diarrhea and bloody stool, and these symptoms were significantly ameliorated by PE treatment. PE administration suppressed DSS-induced expression of proinflammatory enzymes, including cyclooxygenase-2 and inducible nitric oxide synthase as well as cyclin D1, in a dose-dependent fashion. Nuclear factor-kappa B (NF-κB) and signal transducer and activator of transcription 3 (STAT3) are major transcriptional regulators of inflammatory signaling. PE administration significantly inhibited the activation of both NF-κB and STAT3 induced by DSS, while it elevated the accumulation of Nrf2 and heme oxygenase-1 in the colon. In another experiment, treatment of CCD841CoN human normal colon epithelial cells with PE (10 mg/ml) resulted in the attenuation of the tumor necrosis factor-α-induced expression/activation of mediators of proinflammatory signaling. The above results indicate that PE has a preventive potential for use in the management of IBD.

## Introduction

Colorectal cancer has become prevalent not only in Western societies, but also in other parts of the world (Torre et al., 2015). Patients with inflammatory bowel disease (IBD), such as ulcerative colitis and Crohn’s disease, have been at increased risk for developing colorectal cancer (Kim and Chang, 2014). IBD is an illness in which colon becomes inflamed and caused by genetic problems, immune disorders, environmental conditions, and infections (Ananthakrishnan, 2015). Multiple mechanisms, including overproduction of reactive oxygen species (ROS) in the inflamed colonic mucosa, immune response by mucosal inflammatory mediators, and altered intestinal microbiota, are speculated to be involved the pathogenesis of IBD.

Elevated expression and/or activity of cyclooxygenase-2 (COX-2) and inducible nitric oxide synthase (iNOS) have been associated with IBD and inflammation-associated carcinogenesis. COX-2 catalyzes the breakdown of arachidonic acid, thereby producing prostaglandin E_2_, a lipid mediator of inflammation, which promotes tumorigenesis (Wang and Dubois, 2010). iNOS is responsible for production of nitric oxide (NO) derived from L-arginine. Excessive formation of NO causes nitrosative stress which leads to cell cycle disorders, apoptotic cell death, and DNA damage (Aktan, 2004).

Nuclear factor-kappa B (NF-κB) and signal transducer and activator of transcription 3 (STAT3) are representative transcription factors that play prominent roles in inflammatory signaling (Tsatsanis et al., 2006; Witzel et al., 2010). Aberrant overactivation or overexpression of these transcription factors has been frequently observed in inflammation-associated disorders (Danese and Mantovani, 2010). Both cooperate each other, augmenting the inflammatory signal transduction (Bollrath and Greten, 2009; Lee et al., 2009; Grivennikov and Karin, 2010). Under physiological conditions, p65/p50 NF-κB heterodimer remains inactive by forming a complex with inhibitor of κB (IκBα), but becomes activated in response to proinflammatory stimuli through increased phosphorylation and subsequent degradation of IκBα (Hayden and Ghosh, 2004, 2008; Ghosh and Hayden, 2008). This liberates functionally active NF-κB which translocates to nucleus where it regulates the expression of target genes, including those that encode COX-2 and iNOS. STAT3, another transcription factor that plays an important role in inflammation, regulates transcription of genes involved in cell proliferation and survival. Activation of STAT3 is dependent on its phosphorylation at the specific tyrosine residue (Y^705^), which facilitates the dimerization of STAT3. The activated STAT3 dimer then translocates into nucleus to promote the transcription of target genes (Aggarwal et al., 2009; Yue and Turkson, 2009).

Some synthetic anti-inflammatory agents have been considered to have therapeutic potential in the management of IBD, but in many cases their long-term use causes side effects. In this context, it is worthwhile searching for natural sources of anti-inflammatory substances, especially those that have been used for long period of time in herbal medicine or for a culinary purpose, so their safety has been somehow verified. *Perilla frutescens* (L.) Britt. (Lamiaceae) belongs to an annual herb of the mint family. It contains polyphenols which have structural varieties with a large diversity of biological activities (Asif, 2012). Their anti-inflammatory effects have been well-documented (Makino et al., 2001; Ueda and Yamazaki, 2001; Lim et al., 2014; Kwak and Ju, 2015).

Recently, a better understanding of the mechanisms involved in mucosal homeostasis and the occurrence of IBD has been achieved with the advent of animal models of mucosal inflammation. The dextran sulfate sodium (DSS)-induced colitis in mice mimics the pathogenesis of human IBD, and this murine model is hence utilized in preclinical studies for identifying the substances with potential preventive/therapeutic effects on IBD (Wirtz et al., 2007). In the present study, we investigated the possible protective effects of *Perilla frutescens* extract (PE) against DSS-induced colitis and underlying molecular mechanisms, with special focus on the inflammatory signal transduction mediated by NF-κB and STAT3.

## Materials and Methods

### Animals

Four-week-old male ICR mice were purchased from Orient-Bio, Inc. (Seongnam, Republic of Korea). They were acclimated for 1 week with tap water and basal diet under the conventional housing conditions of humidity (50 ± 10%), temperature (24 ± 2°C), and light (12/12 h of light/dark cycle). All studies were approved by the Institutional Animal Care and Use Committee of Seoul National University.

### Induction of DSS-Induced Colitis

**FIGURE 1:**
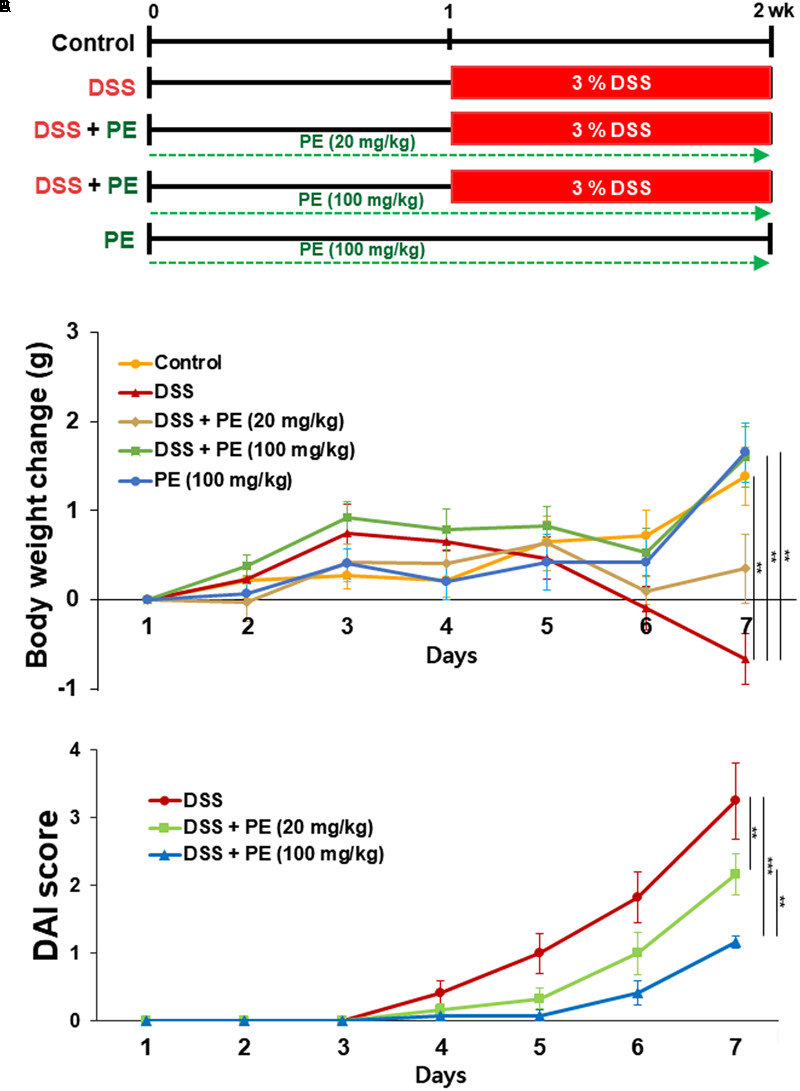
Effects of PE on body weight changes and colitis-associated pathologic symptoms in DSS-treated mice. **(A)** Experimental design for evaluating the effects of PE on DSS-induced mouse colitis. **(B)** Five-week-old male ICR mice were treated with 3% DSS in DDW for 7 days. PE (20 or 100 mg/kg) was dissolved in DDW and given orally 7 days before DSS administration and 7 days together with 3% DSS. Changes in the body weight were measured during the period of DSS treatment. **(C)** DAI as the sum of stool consistency and rectal bleeding was scored 0–4. Data are expressed as means ± SE (*n* = 6 per group). ^∗∗^*p* < 0.01 and ^∗∗∗^*p* < 0.001, one-way ANOVA with *post hoc* Tukey’s test.

DSS (MW of 36,000–50,000) was obtained from MP Biomedicals, LLC (Solon, OH, United States). PE was obtained from Amino Up Chemical Co., Ltd. (Sapporo, Japan). Groups of six mice were treated for 2 weeks in accordance with the experimental design illustrated in **Figure 1A**. Acute colitis was induced by DSS (3%, w/v) given in double distilled drinking water for seven consecutive days. Mice in the PE group received PE (20 or 100 mg/kg) 1 week before and during the DSS administration which was dissolved in double distilled water (DDW) once daily by oral gavage. Mice in control and DSS alone groups were also treated with DDW in the same way to get exposed to an equal stress level. In another experiment, PE was given alone at a daily oral dose of 20 or 100 mg/kg for 2 weeks. At the end of the treatment, mice were sacrificed by cervical dislocation. Their colon tissues were taken out and washed with phosphate-buffered saline (PBS). The tissues were separated into several parts for extraction of protein and mRNA. The distal part was fixed in 10% buffered formalin for immunohistochemical examination, and other parts were frozen in liquid nitrogen immediately and kept at -70°C until use.

### Macroscopic Assessment

Since the beginning of the DSS treatment, the body weight of every mice was measured daily. Rectal bleeding and stool consistency were observed and graded from 0 to 4, depending on the severity of blood and diarrhea (Fukata et al., 2005; Lavi et al., 2010). Disease Activity Index (DAI) was graded as the sum of scores of rectal bleeding and stool consistency. Colorectal parts were photographed, and their length was measured.

### Tissue Lysis and Protein Extraction

Mouse colon tissues were homogenized in ice-cold lysis buffer [150 mM NaCl, 0.5% triton-X 100, 50 mM Tris–HCl (pH 7.4), 20 mM ethylene glycol tetra acetic acid, 1 mM phenylmethylsulfonyl fluoride (PMSF) and ethylenediaminetetraacetic acid (EDTA)-free cocktail tablet] followed by periodical vortex mixing for 30 min at 0°C. Lysates were centrifuged at 18,000 *g* for 15 min at 4°C. The supernatants were collected and stored at -70°C till use.

### Western Blot Analysis

For Western blot analysis, the total protein concentration was quantified by using the bicinchoninic acid protein assay kit (Pierce; Rockford, IL, United States). Lysates (30–50 μg protein) were mixed and boiled in a sodium dodecyl sulfate (SDS) sample buffer for 5 min. The proteins were separated by SDS-polyacrylamide gel electrophoresis and transferred to a polyvinylidene difluoride membrane (Gelman Laboratory; MI, United States). The blots were blocked in 5% fat-free dry milk in Tris-buffered saline containing 0.1% Tween 20 (TBST) for 1 h at room temperature. The membranes were incubated for 12–24 h at 4°C with dilutions of primary antibodies against α-tubulin, actin (Sigma Aldrich, St. Louis, MO, United States), laminB, STAT3, phosphorylated STAT3 (P-STAT3), p65, IκBα, P-IκBα, COX-2, iNOS, CXC chemokine receptor 2 (CXCR2), heme oxygenase-1 (HO-1; Stressgen Biotechnologies Co., San Diego, CA, United States), NF-E2-related factor 2 (Nrf2) and interferon regulatory factor 3 (IRF3; Santa Cruz Biotechnology, Inc., Santa Cruz, CA, United States). The membranes were washed, followed by incubation with 1:4000 dilution of respective horseradish peroxidase (HRP)-conjugated secondary antibody (rabbit, mouse, or goat; Zymed Laboratories; San Francisco, CA, United States) for 2 h, and washed again with TBST. Protein expression was visualized with an enhanced chemiluminescence detection kit (Amersham Pharmacia Biotech; Buckinghamshire, United Kingdom) and LAS-4000 image reader (Fujifilm; Tokyo, Japan) according to the manufacturer’s instructions.

### Fraction of Nuclear and Cytoplasmic Extracts

Cytosolic extracts were obtained from lysates dissolved in hypotonic buffer A [10 mM 4-(2-hydroxyethyl)-1-piperazineethanesulfonic acid (HEPES; pH 7.8), 1.5 mM MgCl_2_, 10 mM KCl, 1 mM dithiothreitol (DTT), 0.1 mM EDTA, and 0.1 mM PMSF] with 0.1% Nonidet P-40 by homogenization and vortex mixing at 10 min intervals for 3 h. After centrifugation at 18,000 *g* for 15 min, the supernatants (the cytosolic extracts) were collected and stored at -70°C until use. Precipitated pellets were suspended in buffer C [20 mM HEPES (pH 7.8), 50 mM KCl, 300 mM NaCl, 0.1 mM EDTA, 1 mM DTT, 0.1 mM PMSF, and 20% glycerol]. After centrifugation at 18,000 *g* for 15 min, the supernatants (the nuclear extracts) were collected and stored at -70°C until use.

### Electrophoretic Mobility Shift Assay

DNA binding activity of NF-κB was measured with electrophoretic mobility shift assay (EMSA). In brief, T4 polynucleotide kinase transferred ^32^P labeled γ-phosphate from ATP to NF-κB oligonucleotide. After purification with a G-50 micro column (GE Healthcare LS; Buckinghamshire, United Kingdom), the γ-^32^P labeled probe was mixed with 10 μg of nuclear extracts and incubation buffer [10 mM Tris–HCl (pH 7.5), 100 mM NaCl, 1 mM DTT, 1 mM EDTA, 4% glycerol, and 0.1 mg/ml sonicated salmon sperm DNA]. All the samples were mixed with 2 μl of 0.1% bromophenol blue loading dye after 50 min incubation and separated on 6% non-denatured polyacrylamide gel in a cold room. Finally, gels were dried and exposed to X-ray films (Agfa Healthcare; Mortsel, Belgium).

### Immunohistochemical Analysis

The dissected colon tissues were prepared for immunohistochemical analysis to measure the expression of COX-2, P-STAT3, and Nrf2. Four-micrometer sections of 10% formalin-fixed and paraffin-embedded tissues were cut on silanized glass slides and deparaffinized three times with xylene and rehydrated through graded alcohol bath. The deparaffinized sections were heated by using microwave and boiled twice for 6 min in 10 mM citrate buffer (pH 6.0) for antigen retrieval. To diminish non-specific staining, each section was treated with 3% hydrogen peroxide and 4% peptone casein blocking solution for 15 min. For the detection of respective protein expression, slides were incubated with COX-2 (Cayman Chemical; Ann Arbor, MI, United States), P-STAT3 (Cell Signaling Technology, Inc.; Beverly, MA, United States) and Nrf2 (Santa Cruz Biotechnology, Inc., Santa Cruz, CA, United States) antibodies at room temperature for 40 min in Tris-buffered saline containing 0.05% Tween 20, and then developed using respective HRP-conjugated secondary antibodies (rabbit, mouse, or goat) EnVision^TM^ System (Dako; Glostrup, Denmark). The peroxidase-binding sites were detected by staining with 3,3′-diaminobenzidine tetrahydrochloride (Dako; Glostrup, Denmark). Finally, counterstaining was performed by using Mayer’s hematoxylin.

### Cell Culture

The CCD841CoN cells were obtained from the American Type Culture Collection (Manassas, VA, United States) and maintained in MEM containing 10% fetal bovine serum and an antibiotic–antimycotic mixture (Gibco BRL; Grand Island, NY, United States) at 37°C with 5% CO_2_ and 95% air. The CCD841CoN cells were treated with PE (10 mg/ml) 1 h before stimulation with 10 ng/ml of tumor necrosis factor-α (TNF-α; R&D Inc.; Minneapolis, MN, United States) dissolved in 0.1% bovine serum albumin in PBS. After TNF-α treatment, CCD841CoN cells were incubated for 1 h and prepped. The cells in the apical slide were harvested and centrifuged at 18,000 *g* for 5 min at 4°C. The pellets were suspended in the cell lysis buffer (Cell Signaling Technology; Beverly, MA, United States). After centrifugation at 18,000 *g* for 15 min, the supernatants were collected and stored at -70°C until use.

### Reverse Transcription PCR

Total RNA was isolated from CCD841CoN cells by using TRIzol^®^ (Invitrogen; Carlsbad, CA, United States) according to manufacturer’s protocol. One microgram of total RNA reverse transcribed with MLV reverse transcriptase at 42°C for 50 min and at 72°C for 15 min. PCR was conducted according to the standard procedures. The primer sequences used were (forward and reverse, respectively): *cox-2*, 5′-GCTGAGCCATACAGCAAATCC-3′ and 5′-GGGAGTCGGGCAATCATCAG-3′; *il-6*, 5′-GTGTGAAAGCAGCAAAGAGGC-3′ and 5′-CTGGAGGTACTCTAGGTATAC-3′; *il-8*, 5′-ATGACTTCCAAGCTGGCCGTGGCT-3′ and 5′-TCTCAGCCCTCTTCAAAAACTTCT-3′; *gapdh*, 5′-AAGGTCGGAGTCAACGGA-3′ and 5′-GCAGTGAGGGTCTCTCTC-3′; *interferon (ifn)*-α, 5′-AGTGAGCTGACCCAGCAGAT-3′ and 5′-AGACAGCCTTGCAGGTCATT-3′; *ifn-β*, 5′-CCCTATGGAGATTGACGGAGA-3′ and 5′-ACCCAGTGCTGGAGAAATTG-3′; *actin*, 5′-AGAGCATAGCCCTCGTAGAT-3′ and 5′-CCCAGAGCAAGAGAGGTATC-3′. Amplified products were analyzed on 2% agarose gel electrophoresis, stained with SYBR^®^ Green (Invitrogen; Carlsbad, CA, United States) and photographed using fluorescence in LAS-4000.

### Statistical Analysis

All values presented are the mean ± standard error (SE) of at least three independent experiments. Statistical significance was determined by the One-way analysis of variance (ANOVA) with Tukey’s *post hoc* test, and *p* < 0.05 was considered to be statistically significant. The bar-graphs were analyzed using GraphPad Prism 7 (GraphPad Software, San Diego, CA, United States).

## Results

### PE Ameliorated Pathological Symptoms in the DSS-Induced Murine Colitis Model

**FIGURE 2:**
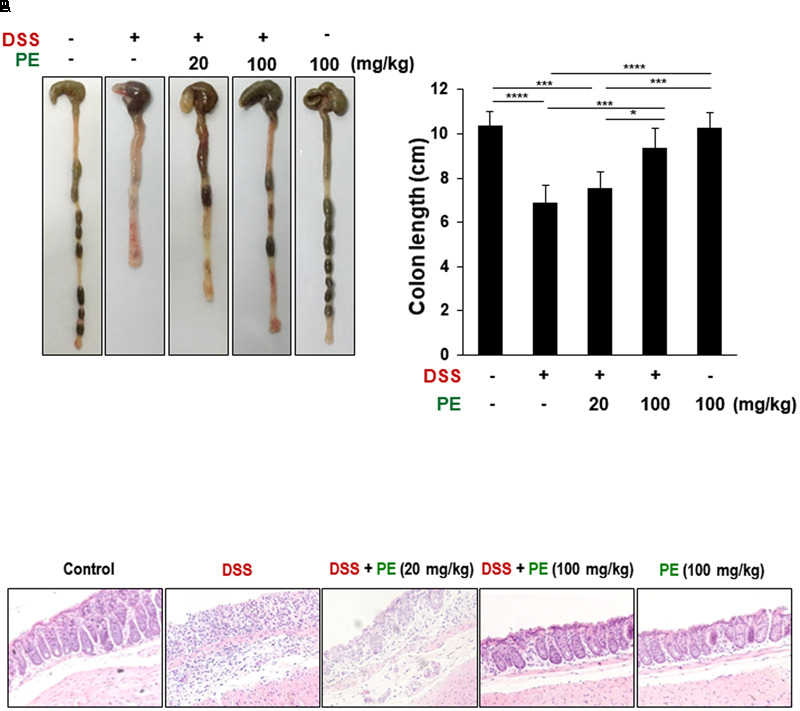
Inhibition of DSS-induced shortening of the colon length and inflammatory cell infiltration by PE. **(A)** The comparison of colon length. Data are expressed as means ± SE (*n* = 6 per group). ^∗^*p* < 0.05, ^∗∗∗^*p* < 0.001, and ^∗∗∗∗^*p* < 0.0001, one-way ANOVA with *post hoc* Tukey’s test. **(B)** The histochemical analysis of colonic mucosa. Collapse and expansion of epithelium were evident in the colon of DSS-treated mice, and these were attenuated by PE administration. Magnification, ×200.

**FIGURE 3:**
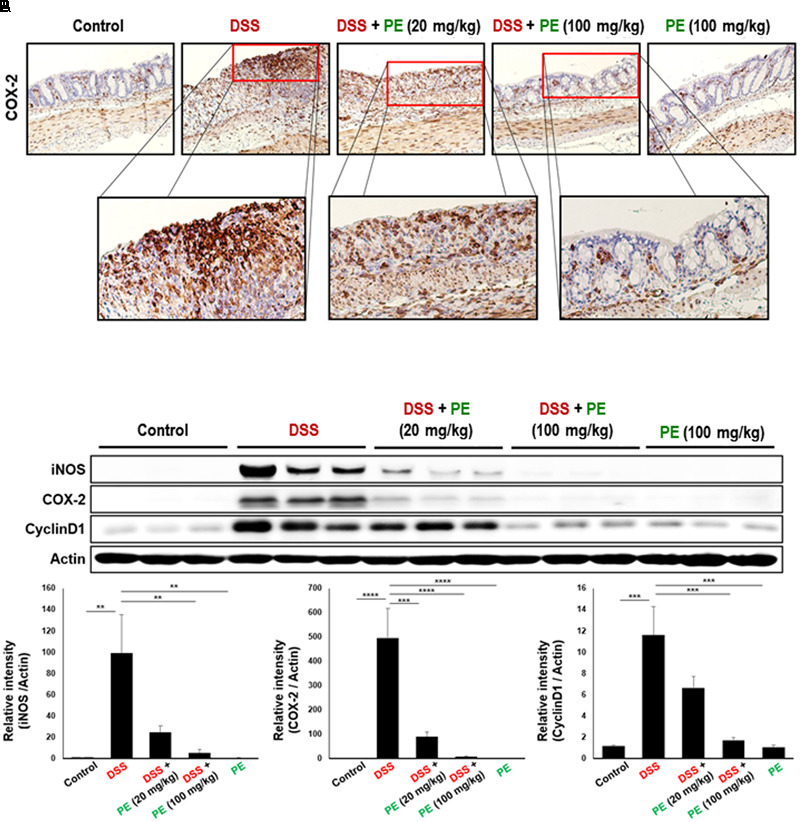
Effects of PE on expression of inflammatory and proliferation markers in DSS-induced mouse colitis. **(A)** Immunohistochemical detection of COX-2. The brown dots represent COX-2 expressing cells. Magnification, ×200. **(B)** Expression of COX-2, iNOS and cyclin D1 in colonic mucosa detected by Western blot analysis. Experimental conditions and other details are described in Section “Materials and Methods.” Data are expressed as means ± SE (*n* = 6 per group). ^∗∗^*p* < 0.01, ^∗∗∗^*p* < 0.001, and ^∗∗∗∗^*p* < 0.0001, one-way ANOVA with *post hoc* Tukey’s test.

The body weight of mice in the DSS group became decreased after the day 3 of DSS treatment compared to the control group. However, oral administration of PE attenuated the loss of body weight (**Figure 1B**). Following the DSS treatment, the DAI score was increased after the day 3. DSS-treated mice exhibited serious symptoms, such as diarrhea and bloody stool. PE treatment reduced the severity of the symptoms (**Figure 1C**). In addition, shrinkage and thickening of colon occurred as a consequence of colitis caused by DSS (**Figure 2A**). Oral administration of PE ameliorated the pathological symptoms of colitis. Inhibitory effects of PE against DSS-induced inflammatory events in mouse colon were also verified by histological, immunohistochemical, and immunoblot analyses. Colorectal tissues from DSS-treated mice showed collapse and expansion of epithelium which were accompanied by infiltration of inflammatory cells (**Figure 2B**) and overexpression of COX-2, iNOS, and cyclin D1 (**Figures 3A,B**). Thus, PE protected the epithelium from DSS-induced colitis as evidenced by attenuation of morphological and biochemical alterations related to inflammatory colonic tissue damage.

### PE Inhibited DSS-Induced Activation of NF-κB and STAT3 Pathways in Mouse Colitis

**FIGURE 4:**
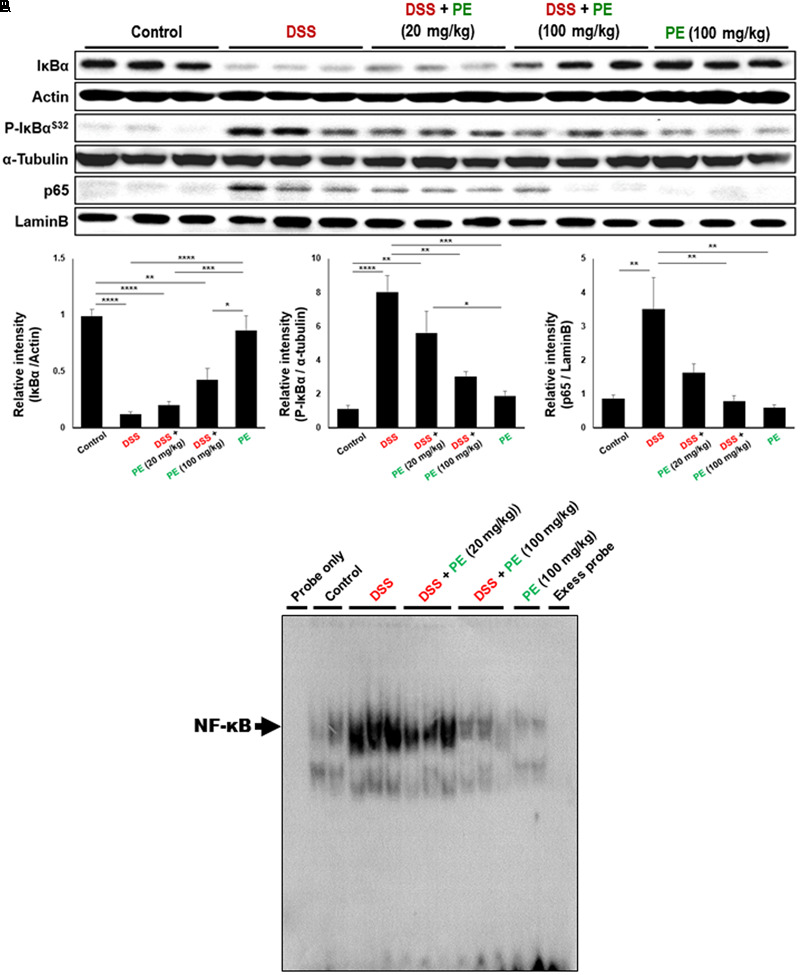
Inhibitory effects of PE on DSS-induced activation of NF-κB signaling. **(A)** Cytosolic extracts and nuclear extracts were subjected to Western blot analysis to detect IκBα (total and phosphorylated) and p65. Data are expressed as means ± SE (*n* = 6 per group). ^∗^*p* < 0.05, ^∗∗^*p* < 0.01, ^∗∗∗^*p* < 0.001 and ^∗∗∗∗^*p* < 0.0001, one-way ANOVA with *post hoc* Tukey’s test. **(B)** DNA binding activity of NF-κB was measured by EMSA as described in Section “Materials and Methods.”

NF-κB is a transcription factor regulating expression of COX-2 and iNOS. It is activated in IBD (Atreya et al., 2008), resulting in disorders of the immune system, elevation of cell survival, and reduction of apoptosis (Aggarwal, 2004). NF-κB activation and translocation into nucleus are dependent on phosphorylation and degradation of IκBα. DSS administration stimulated phosphorylation and subsequent degradation of IκBα, thereby augmenting nuclear translocation of p65, a functionally active subunit of NF-κB. Treatment with PE (100 mg/kg) inhibited the phosphorylation and degradation of IκBα and nuclear migration of p65 (**Figure 4A**). Likewise, the DSS-induced DNA binding activity of NF-κB in colonic mucosa was reduced by the PE administration (**Figure 4B**).

**FIGURE 5:**
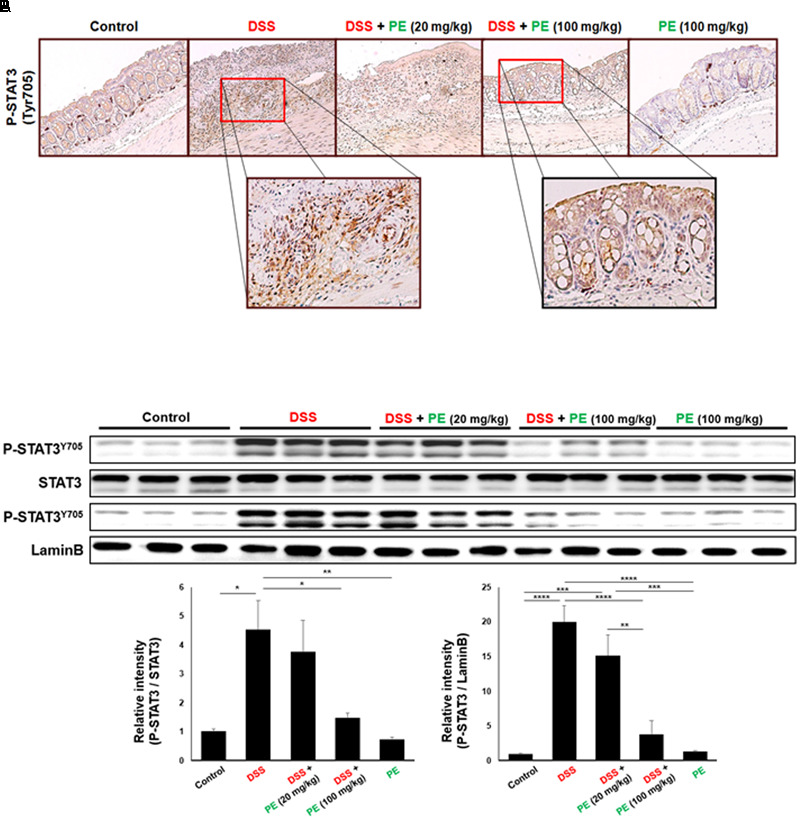
Inhibition of DSS-induced phosphorylation of STAT3 by PE. **(A)** Immunohistochemical detection of STAT3 in the colonic mucosa. Magnification, ×200. **(B)** Protein lysates from total and nuclear extracts of colonic mucosa were used to measure the expression of P-STAT3 as well as STAT3. Data are expressed as means ± SE (*n* = 6 per group). ^∗^*p* < 0.05, ^∗∗^*p* < 0.01, ^∗∗∗^*p* < 0.001, and ^∗∗∗∗^*p* < 0.0001, one-way ANOVA with *post hoc* Tukey’s test.

STAT3 is mainly activated via the interleukin (IL)-6-gp130-JAK axis, leading to phosphorylation on Tyr705. After dimerization, it translocates into nucleus and regulates transcription of target genes by binding to their promoters. The level of P-STAT3 in the colonic tissue was increased by DSS administration (**Figure 5A**), but the phosphorylation and nuclear translocation of STAT3 were significantly inhibited by PE (100 mg/kg) (**Figures 5A,B**).

### PE Attenuated TNF-α-Induced Expression of Inflammatory Factors in Cultured Human Colon Epithelial Cells

**FIGURE 6:**
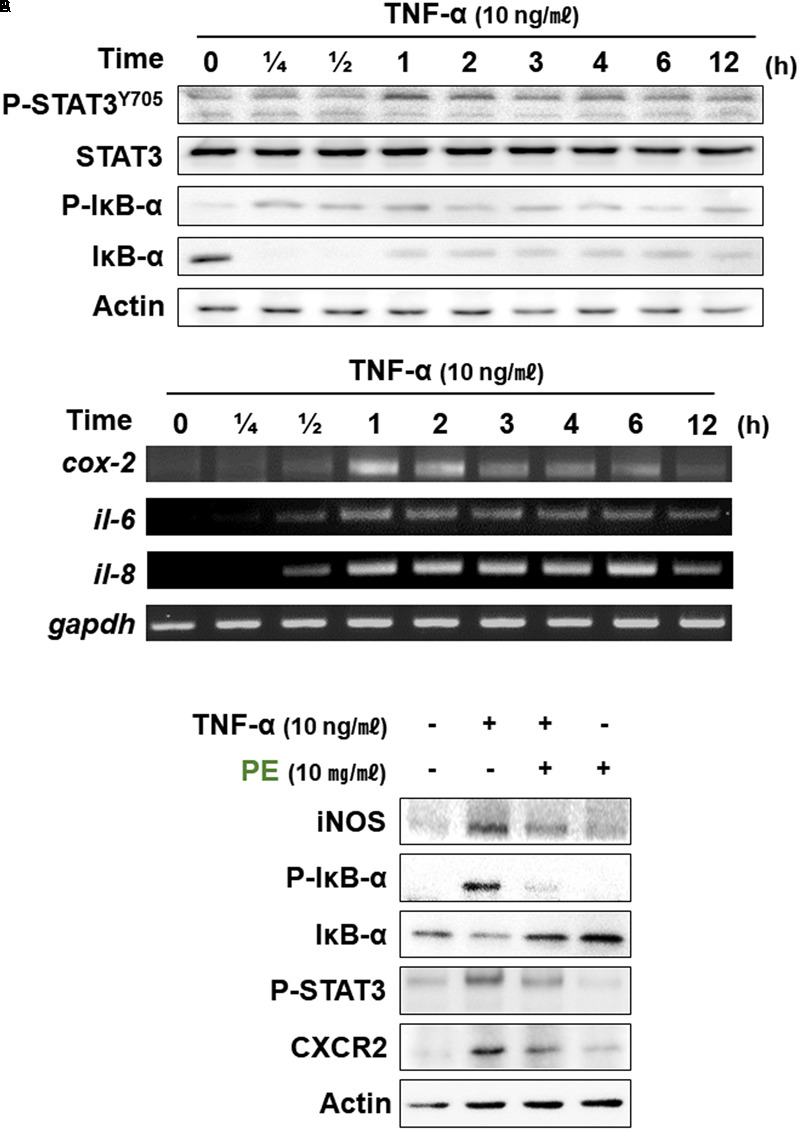
Effects of PE on inflammatory signaling in the CCD841CoN human normal colon cell line stimulated with TNF-α. TNF-α (10 ng/ml) was treated to CCD841CoN for the indicated time periods. The protein **(A)** and mRNA **(B)** levels of inflammatory factors were measured by Western blot assay and RT-PCR, respectively. **(C)** The effects of PE on expression of proinflammatory factors. The cells were treated with TNF-α (10 ng/ml) and PE (10 mg/ml), and whole lysates were subjected to Western blot analysis.

TNF-α is a pleiotropic inflammatory cytokine and a central regulator of inflammation. TNF-α activates NF-κB and MAPK signaling (Bendtzen et al., 2009). CCD841CoN human colon epithelial cells were stimulated with TNF-α (10 ng/ml). Phosphorylation of IκBα and STAT3 was increased in 15 min and 1 h, respectively after TNF-α treatment (**Figure 6A**). IL-6 plays a role not only in acute inflammation, but also in chronic inflammation by changing the nature of leukocytes and modulating some immune cells, such as T- and B-cells. IL-8 is involved in promotion of neutrophil infiltration. TNF-α treatment induced the expression of genes encoding both proteins at 1 h (**Figure 6B**). CXCR2 is a receptor for inflammatory chemokines, such as IL-8. Treatment with PE (10 mg/ml) 1 h prior to TNF-α reduced the expression levels of CXCR2 and some representative mediators involved in inflammatory signaling, such as iNOS, P-IκBα, and P-STAT3 (**Figure 6C**).

### PE Inhibited DSS-Induced Activation of IRF3 Signaling in Mouse Colitis

**FIGURE 7:**
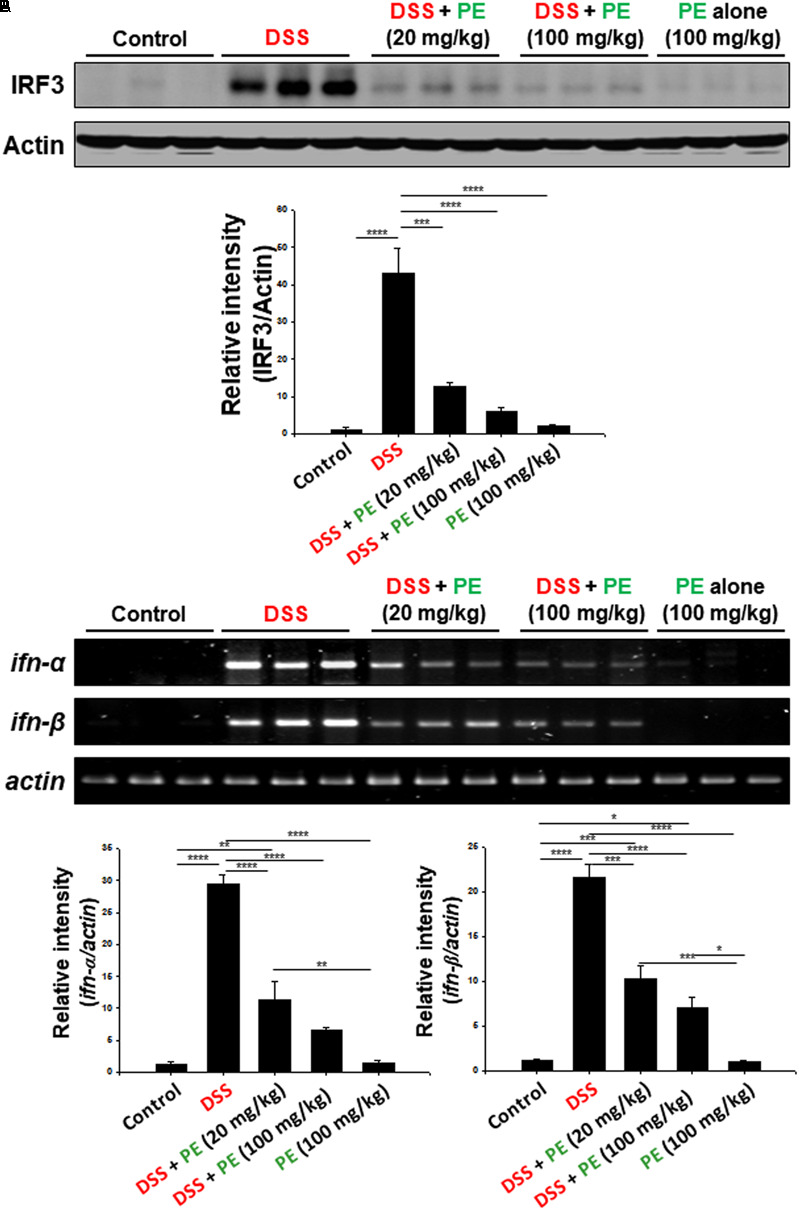
Inhibitory effects of PE on DSS-induced activation of IRF3 signaling in mouse colitis. **(A)** Expression of IRF3 in colonic mucosa was detected by Western blot analysis. **(B)** The mRNA levels of *ifn-α* and *ifn-β* were measured by RT-PCR. Experimental conditions and other details are described in Section “Materials and Methods.” Data are expressed as means ± SE. ^∗^*p* < 0.05, ^∗∗^*p* < 0.01, ^∗∗∗^*p* < 0.001, and ^∗∗∗∗^*p* < 0.0001, one-way ANOVA with *post hoc* Tukey’s test.

Toll-like receptor 4 (TLR4) is an upstream signaling molecule involved in NF-kB activation and pathogenesis of IBD. TLR4 expression has been shown to be upregulated in the colonic mucosa of DSS-induced colitis (Zhang et al., 2014). One of the key mediators of TLR4 signaling is IRF3 that encodes type I interferon (IFN), such as IFN-α and IFN-β. We noticed that there was robust accumulation of IRF3 and expression of *ifn-α* and *ifn-β* in the colonic mucosa of DSS-treated mice (**Figure 7**). PE administration suppressed the expression of IRF3 (**Figure 7A**) and its target genes (**Figure 7B**).

### PE Upregulated the Expression of Nrf2 and its Target Protein HO-1 in the Colon of Mice

**FIGURE 8:**
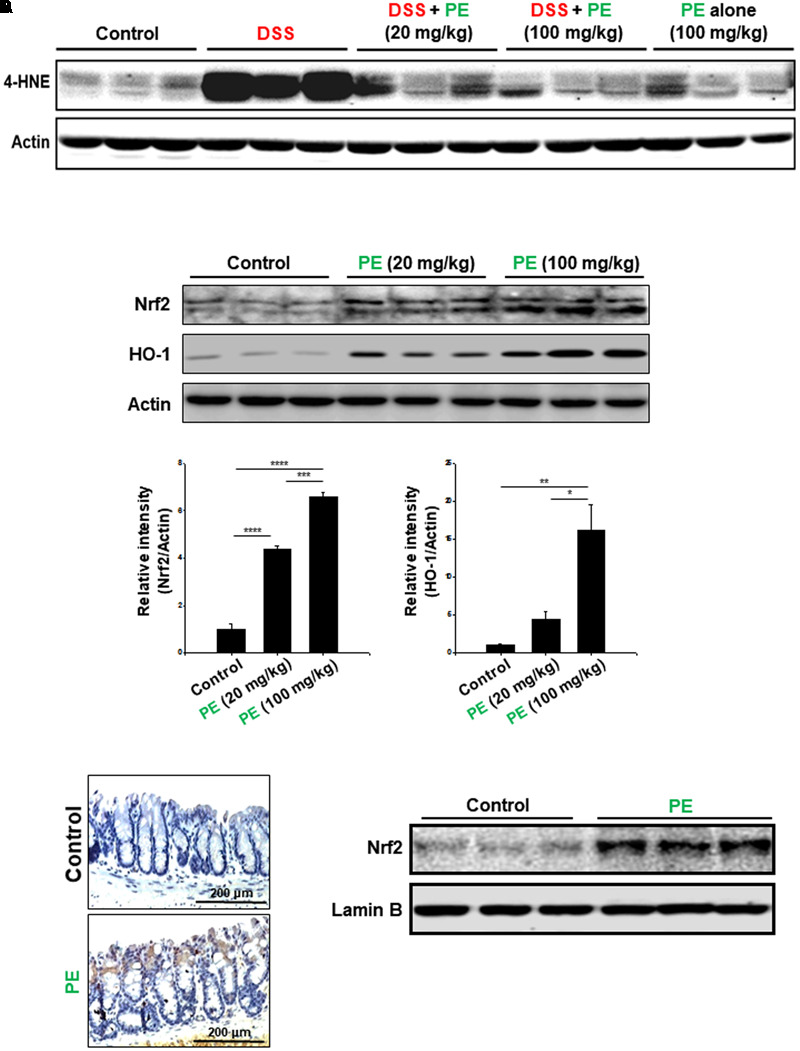
Attenuation of DSS-induced oxidative damage and upregulation of the antioxidant signaling in mouse colon. **(A)** Western blot analysis of 4-HNE-modified protein. **(B)** Effects of PE on expression of Nrf2 and HO-1. To compare the expression of Nrf2 and HO-1, the whole lysates of the colon from vehicle- or PE-treated (20 or 100 mg/kg once a day for 2 weeks) mice were prepared and subjected to Western blot analysis. Data are expressed as means ± SE. ^∗^*p* < 0.05, ^∗∗^*p* < 0.01, ^∗∗∗^*p* < 0.001, and ^∗∗∗∗^*p* < 0.0001, one-way ANOVA with *post hoc* Tukey’s test. **(C)** Immunohistochemical detection of Nrf2 in the colon of mice treated with PE (100 mg/kg) for 2 weeks. **(D)** Effects of PE (100 mg/kg) on nuclear localization of Nrf2 in mouse colon.

DSS administration caused not only inflammation, but also oxidative mucosal damage as revealed by increased accumulation of 4-HNE-modified protein (**Figure 8A**). PE administration attenuated the DSS-induced oxidative stress. Nrf2 is a transcription factor encoded by the *NFE2L2* gene and regulates the expression of many antioxidant enzymes and other stress-responsive proteins, including HO-1 (Moi et al., 1994). PE enhanced the expression of HO-1 as well as Nrf2 (**Figure 8B**). The colonic accumulation of Nrf2 was further verified by immunohistochemical analysis (**Figure 8C**). The nuclear localization of Nrf2 was also enhanced by PE (**Figure 8D**).

## Discussion

Both NF-κB and STAT3 contribute to inflammation and carcinogenesis by cooperating each other (Lee et al., 2009; Grivennikov and Karin, 2010). COX-2 is one of the major target proteins of NF-κB. Dysregulation of cell cycle control plays a key role in carcinogenesis. Cyclin D1 is a key protein that regulates the cell cycle transition from G_1_ to S phase. Cyclin D1 is transcriptionally regulated by STAT3 and required for neoplastic transformation (Leslie et al., 2006). It is interesting to note that COX-2, as a major target of NF-κB, has been found to stimulate human urothelial cell proliferation induced by inorganic arsenic through upregulation of cyclin D1 (Wang et al., 2015). We noticed that cyclin D1 was overexpressed together with COX-2 and another inflammatory enzyme iNOS in DSS-induced colitis, and this was suppressed by PE administration. DSS-induced colitis is also accompanied by oxidative tissue damages. Thus, the colonic mucosa from DSS-treated mice showed elevated accumulation of 4-HNE-modified protein, a biochemical hallmark of oxidative stress. PE upregulated the expression of a prototypic antioxidant enzyme, HO-1 and its master regulator Nrf2. As oxidative stress and inflammation are closely linked, it would be suggested that PE inhibits DSS-induced colitis by fortifying the tissue antioxidant defense system.

While our study was in progress, Urushima et al. (2015) reported that 0.54% PE administered for 7 days simultaneously with 1.5% DSS or for 10 days following 1.5% DSS treatment for 5 days ameliorated pathogenic symptoms of colitis in 8-week-old C57BL/6 female mice while it improved the body weight loss. They found that serum levels of TNF-α, IL-17A, and IL-10 were significantly reduced by co-administration of PE. When PE was given after the DSS treatment, there was a marked elevation of the serum TGF-β level whereas the mRNA expression of TNF-α and IL-17A was significantly down-regulated in the distal colon. Our *in vitro* study revealed that stimulation of human colon epithelial cells with TNF-α resulted in upregulation of some proinflammatory gene expression. IL-6 is a pleiotropic cytokine that is produced at the site of inflammation and plays a key role in the acute inflammatory response (Xing et al., 1998). However, IL-6 is also considered to dictate the transition from acute to chronic inflammation by changing the nature of leukocytes. In addition, IL-6 exerts stimulatory effects on T- and B-cells, thus favoring chronic inflammatory responses (Gabay, 2006). IL-8 is a chemokine produced by macrophages and other cell types, such as epithelial cells, and attracts and activates neutrophils in inflamed sites (Bickel, 1993). CXCR2 is a receptor for IL-8, and their interaction triggers neutrophil infiltration into epithelial tissues, making them inflamed (Katoh et al., 2013). We found that PE treatment to human colon epithelial cells inhibited the expression of TNF-a-induced CXCR2 expression. Taken all these into account, the protective effect of PE against DSS-induced colitis is attributable, at least in part, to its modulation of immune cell functions. Our preliminary studies have revealed that PE treatment to murine macrophages inhibits the LPS-induced expression of proinflammatory cytokines and enhances their phagocytic activity in terms of engulfing apoptotic neutrophils (DD Park et al., unpublished observation).

The transcription factor, IRF3 plays an indispensable role in innate host immune response to microbial infection. IRF3 has been shown to have an anti-inflammatory function (Carrigan et al., 2010), but a recent study also reveals that it can mediate the pro-inflammatory signals (Kumari et al., 2016). In our present study, DSS treatment resulted in pronounced induction of IRF3 and two of its principal target genes, *ifn-α* and *ifn-β*, and this was repressed by PE administration. It has been reported that rebamipide, used in Japan for the treatment of gastric ulcers and other gastrointestinal disorders, inhibited the DSS-induced IRF3 upregulation (Ogasawara et al., 2011). Myeloid derived suppressor cells (MDSCs) are immature myeloid cells that comprise a heterogeneous immune cell population. The proportion of MDSCs increases in inflammation and infection. Development of solid cancer depends on escape from host immune surveillance. MDSCs play pivotal roles in suppression of host immune responses through multiple mechanisms (Wesolowski et al., 2013; Katoh and Watanabe, 2015). STAT3 activation promotes expansion of MDSCs, providing a permissive immune environment necessary for the growth of malignant cells. Therefore, STAT3 and related pathways may serve as a target for changing the tumor immunologic microenvironment to benefit cancer immunotherapies (Lee et al., 2011). It would be worthwhile investigating the effects of PE and it active constituents on the recovery of immune surveillance in inflammation-associated intestinal carcinogenesis.

PE extract (150 mg, twice a day, *p.o.*) has been reported to improve gastrointestinal discomfort in a double-blind, randomized, placebo-controlled study (Buchwald-Werner et al., 2014). PE contains many polyphenols and some of them have strong anti-inflammatory and antioxidant effects (Zhou et al., 2014). Major components of PE are caffeic acid, rosmarinic acid, luteolin, and apigenin. Luteolin suppressed production of proinflammatory cytokines, such as TNF-α, IL-1β, IL-6, and IL-17A in cultured monocytes derived from the 8-week-old wild-type C57/BL6 female mice. Apigenin also suppressed secretion of IL-17A while it increased the anti-inflammatory cytokine IL-10, whereas rosmarinic acid increased the regulatory T cell population (Urushima et al., 2015). Rosmarinic acid has recently been shown to suppresses DSS-induced colitis which is attributed to its dual inhibition of NF-κB and STAT3 activation (Jin et al., 2017).

**FIGURE 9:**
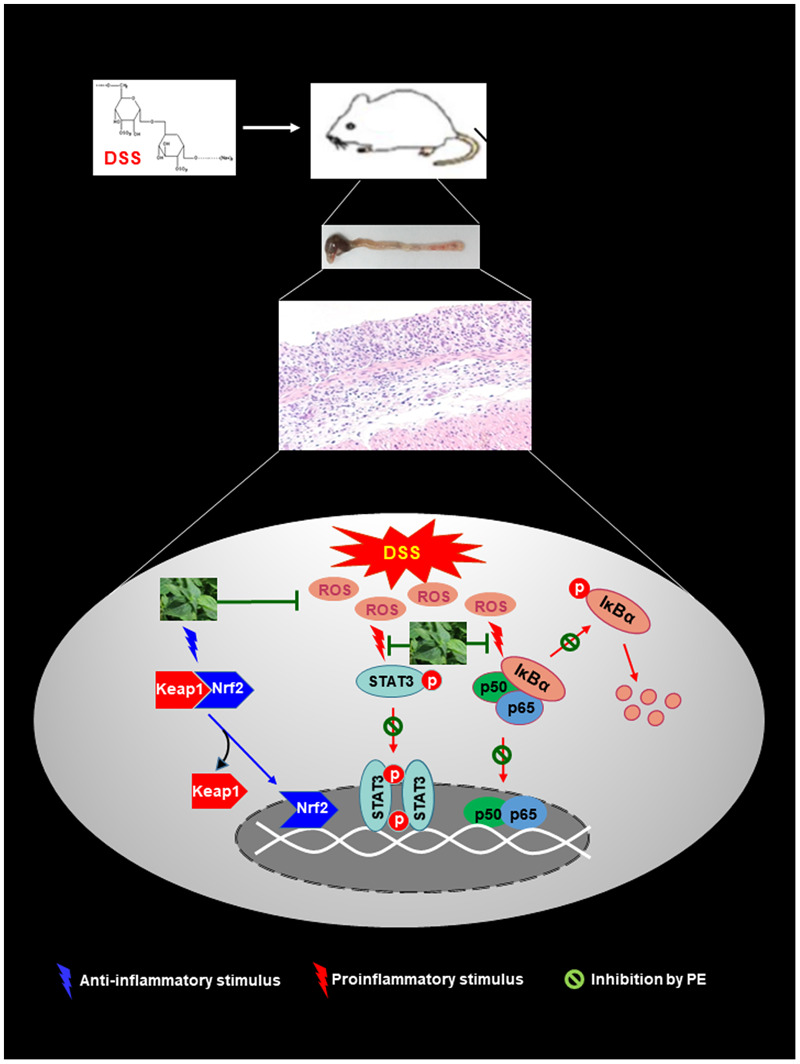
Proposed scheme illustrating the biochemical mechanisms underlying protective effects of PE against DSS-induced mouse colitis. PE inhibits pro-inflammatory signaling mediated by NF-kB and STAT3 while it activates the Nrf2-induced antioxidant signaling.

In summary, PE administration ameliorated the experimentally induced colitis in mice. The protective effects of PE appear to be associated with blockade of proinflammatory signaling mediated by NF-κB and STAT3 and potentiation of Nrf2-mediated antioxidant defense as schematically illustrated in **Figure 9**. These findings suggest that PE can be considered for use in the management of IBD, and further clinical studies will be necessary to evaluate its safety and therapeutic efficacy and to identify an active principle.

## Author Contributions

DDP conducted a majority of experiments and wrote the manuscript; H-WY, XZ, SeuHK, D-HK, and S-JK helped experiments and provided experimental materials and techniques; SeoHK conducted experiments required for revision; H-KN advised the experiments and participated in discussion; AS and TM supplied the *Perilla frutescens* and provided relevant information for revision; Y-JS guided the entire study and edited the manuscript.

## Conflict of Interest Statement

The authors declare that the research was conducted in the absence of any commercial or financial relationships that could be construed as a potential conflict of interest.
